# Thyroid hormone signaling is associated with physical performance, muscle mass, and strength in a cohort of oldest-old: results from the Mugello study

**DOI:** 10.1007/s11357-020-00302-0

**Published:** 2020-11-21

**Authors:** Angelo Di Iorio, Roberto Paganelli, Michele Abate, Giovanni Barassi, Alex Ireland, Claudio Macchi, Raffaele Molino-Lova, Francesca Cecchi

**Affiliations:** 1 Department of Medicine and Science of Aging, Laboratory of Clinical Epidemiology and Aging, University “G. d’Annunzio” Chieti, University Centre of Sports Medicine, Viale Abruzzo 322, Chieti Scalo, Italy; 2 Thermal Medicine Center of Castelnuovo della Daunia, Foggia, Italy; 3 Department of Life Sciences, Musculoskeletal Science and Sports Medicine Research Centre, Manchester Metropolitan University, John Dalton Building, Chester Street, Manchester, M1 5GD UK; 4 Department of Experimental and Clinical Medicine, Università Di Firenze, Largo Brambilla 3, 50100 Florence, Italy; 5 IRCCS Fondazione Don Carlo Gnocchi, Via di Scandicci 269, 50143 Florence, Italy

**Keywords:** Thyroid hormone signaling, Aging, Oldest-old, Physical performance, Muscle mass, Muscle strength, Rehabilitation

## Abstract

Thyroid hormones (THs) play a crucial role in the homeostasis of muscle function, such as myogenesis and energy metabolism, suggesting that the thyroid may be also involved in the entropic processes of muscle aging. The aim of the present study is to evaluate the effect of TH signaling on physical performance, muscle mass, and strength in a cohort of community-dwelling oldest-old subjects (> 90 years). The study population was selected in a rural area of central Italy (Mugello, Tuscany), and the design was cross-sectional. Four hundred seventy-five subjects (130 males and 345 females) were enrolled, representing about 65% of all the nonagenarians living in the Mugello area. After adjusting for multiple confounding factors (sex, age, diabetes, and levothyroxine administration), the lowest quartile of FT3/FT4 ratio distribution showed lower physical performance compared to the other quartiles (*β* ± SE: − 0.49 ± 0.12; *p* < 0.001), whereas the highest quartile of FT3/FT4 ratio was associated with higher skeletal muscle index (*β* ± SE: 1.11 ± 0.42; *p* = 0.009). In addition, the lowest quartile of FT4 showed a statistically significant higher handgrip strength (*β* ± SE: 1.78 ± 0.68; *p* = 0.009) compared to all other quartiles. This study demonstrates that nonagenarians with higher FT3/FT4 ratios had better preserved muscle function, therefore successfully overcoming the imbalance of homeostatic and entropic processes involved in muscle aging. However, we could not establish a cause-effect relationship due to the cross-sectional design of the study.

## Introduction

In 2050, it is expected that one in six people worldwide will be aged 65 years or over [1]. Aging is a natural process that may present a decline in the functional status of patients and is a common cause of subsequent disability [2]. Musculoskeletal, neurological, circulatory, or sensory conditions can lead to a decrease in physical function [3–5].

Longitudinal studies have demonstrated that fat mass increases, whereas muscle mass, strength, and physical performance progressively decline during aging, even if with different rates of progression [6, 7]. Several factors have been hypothesized as determinants of those changes; among them are diseases, physical inactivity, inflammation, malnutrition, reduced metabolic rate, and hormonal imbalance [8–10]. All these processes may occur as part of a common pathway or may be interrelated, for example, aging people with a lower metabolic rate have a significantly lower level of multiple morbidities, and they are healthier than those with a higher metabolic rate [11]. If reduced metabolism is a protective mechanism, it may explain why people who have lower free triiodothyronine (FT3) and free thyroxine (FT4) and also have a lower FT3/FT4 ratio are still alive at an older age [12, 13]. The increase of FT3 production from FT4 due to the action of TSH becomes less pronounced with aging and may be completely lost in the elderly [12]. This could reflect either reduction in deiodinase (DIO) activity with age, and/or development of thyroid hormone resistance with increasing age [14]. In an euthyroid population (40–69 years) from Korea, low serum FT3/FT4 was found to be a reliable index for low muscle mass and impaired physical performance [15]. Similar results were found in a cohort of centenarians and offspring, where THs levels predict disability, mortality, and cognitive level [13]; in another recent work in the same cohort THs, specifically FT3/FT4 ratio, levels were inversely associated with frailty score [16].

Moreover, thyroid hormone off-label supplementation, in euthyroid subjects, was tested in different conditions [17], but levothyroxine (LT4) does not reverse the nonspecific symptoms [18].

The aim of the present study is to evaluate the effect of thyroid hormone signaling on physical performance, muscle mass, and strength in a cohort of community-dwelling oldest-old subjects (> 90 years).

## Methods

The design of the Mugello study has been described in detail elsewhere [19]. Briefly, the study was designed and conducted by the Department of Experimental and Clinical Medicine, University of Florence, Italy, and by the Don Carlo Gnocchi Foundation (Florence, Italy). The study was performed in the Mugello area, a widespread valley northeast of Florence, in the Tuscany region. The study was designed as a cross-sectional survey, and data were collected in 2009.

### Samples

Of the 475 subjects (130 men and 345 women) enrolled in the study, representing approximately 65% of the nonagenarians living in the Mugello area, 417 subjects who consented to donate a blood sample and had all the variables of interest were included. A small group of institutionalized nonagenarians were also included. There was no exclusion criterion. The study protocol, which complied with the principles of the Declaration of Helsinki on clinical research involving human subjects, was approved by the Institutional Review Board. All participants, or proxies, signed the informed consent form to be included in the study.

### Home interview

A trained interviewer investigated family, medical, and medication history. In particular, family history of cardiovascular, cerebrovascular, and respiratory diseases; cancer; and dementia was obtained. Past and recent medical history was recorded using a semi-structured questionnaire.

### Comprehensive geriatric assessment

Some specific geriatric items, such as functional independence, physical activity level, quality of life, mood, sleep quality, and falls, were assessed.

### Clinical assessment

The interview was followed by a general physical and clinical examination with special attention to general and neurological condition, by a semi-structured questionnaire. Anthropometric measures, such as weight, height, ulna length, lower limb length (from the anterior–superior iliac spine to the internal malleolus), abdominal and hip circumference, as well as arm, thigh, and leg circumference, were also recorded.

### Body composition

Body composition was assessed by using Body Impedance Assessment (BIA) (EFG, Akern, Italy). BIA measures the opposition of body tissues to the flow of a small (less than 1 mA) alternating current by providing two values (resistance and reactance). Muscle mass was calculated using the Sergi equation [20]. Skeletal muscle index (SMI) was obtained from the standardization of the absolute skeletal muscle mass per squared meters, calculated through the Janssen and colleagues equation [21].

### Physical performance

The short physical performance battery (SPPB) based on the lower extremity performance tests used in the Established Populations for the Epidemiologic Studies of the Elderly (EPESE) was used here to summarize lower extremity performance [22]. The SPPB consisted of walking speed, ability to stand from a chair, and ability to maintain balance in progressively more challenging positions. A score ranging from zero, representing inability to do the test, to one, representing the highest level of performance, was calculated from the three physical performance tasks. The score in every timed-task was calculated as the inverse of the percentage, where the worst time is the unity. Lastly, the three measures were added to create a summary physical performance measure ranging from zero (worst) to three (best). This approach for the calculation of the performance was needed since specific age adjusted cutoff points are not available; therefore the classic way to calculate the SPPB score could produce left-skewed data.

### Handgrip test

Handgrip strength was measured using a handheld dynamometer (Hydraulic Dynamometer, RO+TEN, Italy). Participants were asked to perform the task twice with each hand. The average of the best result obtained with each hand was used for these analyses.

### Blood collection

Finally, about 1 week after the clinical assessment, a nurse collected venous blood samples for routine and special laboratory tests. Serum and plasma aliquots were also stored at − 80° in the biologic bank.

### Laboratory tests

Serum levels of thyroid-stimulating hormone (TSH) were measured using an enzyme-linked immunosorbent assay (ELISA), with the WHO First International Reference Standard, and a sensitivity of 0.08 μg/mL; a colorimetric competitive immunoassay was used to measure T3 and T4 with purified protein standard and polyclonal anti-T3 and anti-T4 (Diesse Diagnostica Senese, Chorus fT3, fT4, TSH, Monteriggioni, Siena, Italy); serum creatinine level was assessed using a standard creatinine Jaffe method (Roche Diagnostics, GmbH, Mannheim, Germany), which had an inter-assay coefficient below 2.5%.

### Statistical analysis

Cross-sectional characteristics were compared between quartiles of distribution of FT3, FT4, TSH, and FT3/FT4 ratio, for all the variables of interest; differences among quartiles were evaluated using analysis of variance for continuous variables and *χ*^2^ test analyses for dichotomous or categorical variables.

To analyze whether statistically significant differences could be found in the mean values of SPPB score, handgrip test, and muscle mass/height squared, between subjects in the THs quartiles of distribution, linear regression models were used, adjusting for age and sex.

Lastly, to evaluate the independent but concurrent role of different markers of thyroid signaling hormones on physical performance, strength, and muscle mass, three separate linear regression models were analyzed. Only dummy quartile derived variables were considered, and the different models were adjusted for age, sex, diabetes, and levothyroxine therapy, and variables were not excluded from the model even if they did not reach a statically significant level.

The backward selection was made visually on the *p* value basis. AIC, *R*^2^, and RSSE goodness of fit tests were assessed to discriminate variables better describing the different associations in the parsimonious model [23].

Analyses were conducted using SAS 9.4 (SAS Institute Inc., Cary, NC, USA).

## Results

Four hundred seventeen subjects were enrolled in this study; 111 (26.62%) were males, and the mean age of the total population was 93.00 ± 3.16 years, with slightly but significantly younger age in males (*M* = 92.37 ± 2.66 years; *F* = 93.22 ± 3.30; *p* = 0.01).

Basic participant characteristics were found to be similar when the variables were analyzed according to TSH levels ranked in quartiles, the only exception being the ratio of muscle mass/height squared ratio, which increased from the lowest quartile to the highest one (*p* = 0.04) (Table 1).

**Table 1:** Descriptive of the population enrolled in the Mugello study according to quartiles of TSH (μIU/mL) distribution

	TSH < 0.79	TSH 0.80–1.25	TSH 1.26–2.11	TSH > 2.11	
	106	103	104	104	*p* trend
Sex male *n* (%)	28 (26.4)	34 (33.3)	27 (26.0)	22 (21.2)	0.27
Age (yy)	93.03 ± 3.40	92.87 ± 3.05	92.88 ± 3.36	93.22 ± 2.85	0.68
Weight (kg)	60.53 ± 11.48	61.62 ± 12.54	62.77 ± 12.78	62.44 ± 13.46	0.22
Height (m)	1.56 ± 0.08	1.58 ± 0.10	1.57 ± 0.10	1.57 ± 0.08	0.74
Smoke (actually)	26 (24.5)	35 (34.3)	29 (27.9)	26 (25.0)	0.15
Diabetes	11 (10.3)	12 (11.8)	20 (19.2)	14 (13.5)	0.25
Cardiovascular diseases *n* (%)	49 (46.3)	46 (45.1)	48 (46.2)	47 (45.2)	0.97
Respiratory diseases *n* (%)	15 (14.2)	16 (15.7)	15 (14.4)	15 (14.4)	0.99
Cerebrovascular diseases *n* (%)	14 (13.2)	26 (25.5)	21 (25.5)	24 (23.1)	0.20
Dementia *n* (%)	15 (14.2)	11 (10.8)	11 (10.8)	13 (12.5)	0.82
Oncological diseases *n* (%)	53 (50.0)	65 (63.7)	53 (51.0)	55 (52.9)	0.52
SPPB (0–3)	1.15 ± 1.07	1.24 ± 1.10	1.17 ± 1.03	1.12 ± 1.05	0.74
Handgrip (kg)	13.20 ± 6.73	13.88 ± 8.12	13.97 ± 6.38	12.49 ± 7.06	0.56
Levothyroxine	2 (1.9)	4 (3.9)	1 (0.9)	11 (10.6)	0.003
Muscle/height^2^ (kg/m^2^)	11.51 ± 3.38	11.76 ± 2.72	12.01 ± 3.01	12.52 ± 3.13	0.04
Fat mass (kg)	15.89 ± 8.50	14.50 ± 8.22	16.29 ± 10.53	16.60 ± 9.75	0.44
FT3 (pg/m)	2.98 ± 0.69	2.86 ± 0.34	2.79 ± 0.39	2.76 ± 0.45	0.001
FT4 (ng/dL)	0.95 ± 0.29	0.91 ± 0.19	0.85 ± 0.17	0.81 ± 0.22	0.001
TSH (μIU/mL)	0.50 ± 0.23	1.01 ± 0.14	1.62 ± 0.25	5.42 ± 9.87	0.001
FT3/FT4 ratio	3.32 ± 1.03	3.29 ± 0.81	3.39 ± 0.80	3.65 ± 1.23	0.01

*SPPB* short physical performance battery. Values are presented as mean ± standard deviation for continuous variables, and *n* (%) for categorical variables

A similar trend was found for distribution in quartiles of the values of serum FT3 (Table 2). In this case too, muscle mass/height squared ratio showed a linear increase through the different quartiles (St*β* ± SE = 0.11 ± 0.05). An increase of the SPPB score from lowest through highest quartile was also observed (St*β* ± SE = 0.17 ± 0.05).

**Table 2:** Descriptive of the population enrolled in the Mugello study according to FT3 (pg/m) quartiles of distribution

	FT3 < 2.56	FT3 2.57–2.78	FT 32.79–3.05	FT3 > 3.05	
	103	100	114	100	*p* trend
Sex male	24 (23.3)	28 (28.0)	31 (27.2)	28 (28.0)	0.85
Age (yy)	93.37 ± 3.73	93.12 ± 3.12	92.75 ± 2.82	92.80 ± 2.92	0.13
Weight (kg)	62.89 ± 13.31	61.77 ± 12.19	60.80 ± 12.38	62.28 ± 12.57	0.61
Height (m)	1.57 ± 0.09	1.58 ± 0.09	1.57 ± 0.09	1.57 ± 0.10	0.33
Smoke (actually)	23 (22.3)	31 (31.0)	35 (30.7)	24 (24.0)	0.07
Diabetes	20 (19.4)	14 (14.0)	16 (14.0)	7 (6.9)	0.08
Cardiovascular diseases	54 (52.4)	44 (44.0)	47 (41.2)	45 (45.0)	0.47
Respiratory diseases	13 (12.6)	13 (13.0)	21 (18.4)	15 (15.0)	0.71
Cerebrovascular diseases	18 (17.5)	24 (24.0)	16 (14.0)	27 (27.0)	0.07
Dementia	13 (12.6)	12 (12.0)	13 (11.4)	12 (12.0)	0.98
Oncological diseases	52 (50.5)	56 (56.0)	66 (57.9)	53 (53.0)	0.85
SPPB (0–3)	0.87 ± 0.97	1.06 ± 1.10	1.35 ± 1.07	1.37 ± 1.01	< 0.001
Handgrip (kg)	12.92 ± 7.95	13.57 ± 6.07	13.20 ± 6.84	13.91 ± 7.39	0.44
Levothyroxine	9 (8.7)	6 (6.0)	3 (2.6)	0	0.07
Muscle/height^2^ (kg/m^2^)	11.34 ± 2.72	11.98 ± 2.49	12.06 ± 3.44	12.46 ± 3.44	0.04
Fat mass (kg)	17.11 ± 9.87	15.87 ± 9.40	14.73 ± 8.30	16.12 ± 10.03	0.36
FT3 (pg/m)	2.35 ± 0.17	2.67 ± 0.06	2.92 ± 0.08	3.45 ± 0.56	< 0.001
FT4 (ng/dL)	0.85 ± 0.21	0.88 ± 0.22	0.86 ± 0.18	0.93 ± 0.28	0.02
TSH (μIU/mL)	3.51 ± 9.75	1.63 ± 1.23	1.87 ± 3.40	1.52 ± 1.37	0.01
FT3/FT4 ratio	2.98 ± 1.08	3.22 ± 0.77	3.53 ± 0.78	3.92 ± 1.05	< 0.001

*SPPB* short physical performance battery. Values are presented as mean ± standard deviation for continuous variables, and *n* (%) for categorical variables

Table 3 reports the clinical characteristics of the participants according to the FT4 quartile of distribution. A linear trend in age and weight (*p* = 0.007 and *p* = 0.02, respectively) was found through the quartiles. A linear relationship was also present for physical performance (SPPB score St*β* ± SE = − 0.16 ± 0.04) and muscle strength (handgrip St*β* ± SE = 0.14 ± 0.04).

**Table 3:** Descriptive of the population enrolled in the Mugello study according to FT4 (ng/dL) quartiles of distribution

	FT4 < 0.73	FT4 0.74–0.86	FT4 0.75–1.00	FT4 > 1.00	
	102	107	105	103	*p* trend
Sex male	29 (28.43)	21 (19.63)	32 (30.48)	29 (28.16)	0.29
Age (yy)	92.57 ± 2.81	92.76 ± 2.93	92.93 ± 3.18	93.76 ± 3.59	0.007
Weight (kg)	64.96 ± 13.18	60.99 ± 11.64	61.14 ± 12.78	60.52 ± 12.43	0.02
Height (m)	1.58 ± 0.09	1.55 ± 0.08	1.58 ± 0.11	1.57 ± 0.09	0.78
Smoke (actually)	30 (29.41)	30 (28.04)	28 (26.67)	25 (24.27)	0.70
Diabetes	13 (12.8)	9 (8.4)	13 (12.3)	22 (21.4)	0.06
Cardiovascular diseases	42 (41.18)	52 (48.60)	52 (49.52)	44 (42.72)	0.55
Respiratory diseases	18 (17.65)	22 (20.56)	14 (13.33)	8 (7.77)	0.04
Cerebrovascular diseases	19 (18.63)	23 (21.50)	19 (18.10)	24 (23.30)	0.78
Dementia	10 (9.80)	19 (17.76)	11 (10.48)	10 (9.71)	0.18
Oncological diseases	60 (58.82)	55 (51.40)	55 (52.38)	57 (55.34)	0.74
SPPB score (0–3)	1.43 ± 1.03	1.27 ± 1.04	1.06 ± 1.10	0.90 ± 1.00	< 0.001
Handgrip (kg)	14.84 ± 7.61	13.22 ± 6.64	13.33 ± 7.83	12.05 ± 5.85	0.01
Levothyroxine	4 (3.96)	5 (4.8)	2 (1.9)	7 (6.8)	0.37
Muscle/height^2^ (kg/m^2^)	12.72 ± 3.16	12.16 ± 2.61	11.54 ± 3.49	11.81 ± 3.00	0.23
Fat mass (kg)	17.35 ± 10.32	15.27 ± 8.06	15.94 ± 9.32	15.20 ± 9.66	0.25
FT3 (pg/m)	2.80 ± 0.50	2.83 ± 0.39	2.83 ± 0.38	2.93 ± 0.65	0.06
FT4 (ng/dL)	0.64 ± 0.09	0.79 ± 0.04	0.93 ± 0.04	1.16 ± 0.22	< 0.001
TSH (μIU/mL)	3.94 ± 10.18	1.73 ± 1.41	1.40 ± 0.99	1.51 ± 1.91	0.001
FT3/FT4 ratio	4.48 ± 1.11	3.59 ± 0.52	3.05 ± 0.43	2.55 ± 0.50	< 0.001

*SPPB* short physical performance battery. Values are presented as mean ± standard deviation for continuous variables, and *n* (%) for categorical variables

Finally, Table 4 describes the clinical characteristics of the population according to FT3/FT4 ratios subdivided in quartiles. The subjects classified in the lowest quartile were older compared to all other groups, and consequently, dementia and incontinence were slightly more prevalent (*p* = 0.04; *p* = 0.05, respectively); for the same reason, subjects in this quartile displayed a lower physical performance (*p* < 0.001), strength (*p* = 0.04), and muscle mass/height squared ratio (*p* = 0.04) compared to other quartiles.

**Table 4:** Descriptive of the population enrolled in the Mugello study according to FT3/FT4 ratio quartiles of distribution

	FT3/FT4 < 2.75	FT3/FT4 2.76–3.32	FT3/FT4 3.33–3.84	FT3/FT4 > 3.84	
	102	107	105	103	*p* trend
Sex male	25 (23.81)	35 (32.71)	28 (27.18)	23 (22.55)	0.34
Age (yy)	94.28 ± 3.89	92.64 ± 2.98	92.48 ± 2.48	92.61 ± 2.78	< 0.001
Weight (kg)	60.64 ± 13.39	61.68 ± 11.22	62.32 ± 13.05	62.96 ± 12.68	0.18
Height (m)	1.58 ± 0.09	1.57 ± 0.10	1.57 ± 0.09	1.57 ± 0.08	0.77
Smoke (actually)	20 (19.05)	33 (30.84)	31 (30.10)	29 (28.43)	0.12
Diabetes	26 (24.8)	14 (13.0)	5 (4.9)	12 (11.8)	0.003
Cardiovascular diseases	48 (45.71)	54 (50.47)	47 (45.63)	41 (40.20)	0.51
Respiratory diseases	9 (8.57)	16 (14.95)	15 (14.56)	22 (21.57)	0.10
Cerebrovascular diseases	22 (20.95)	22 (20.56)	21 (20.39)	20 (19.61)	0.97
Dementia	17 (16.19)	5 (4.67)	14 (13.59)	14 (13.73)	0.04
Oncological diseases	51 (48.57)	60 (56.07)	57 (55.34)	59 (57.84)	0.55
SPPB score (0–3)	0.70 ± 0.93	1.20 ± 1.12	1.31 ± 1.02	1.47 ± 1.01	< 0.001
Handgrip (kg)	11.29 ± 6.13	13.98 ± 7.96	14.56 ± 6.75	13.51 ± 7.01	0.04
Muscle (kg)	29.19 ± 8.81	29.20 ± 7.40	28.59 ± 9.08	32.21 ± 9.19	0.08
Muscle/height^2^ (kg/m^2^)	11.57 ± 3.06	11.89 ± 2.79	11.64 ± 3.21	12.78 ± 3.19	0.04
Fat mass (kg)	15.89 ± 9.98	14.75 ± 7.86	17.86 ± 9.97	15.26 ± 9.46	0.78
FT3 (pg/m)	2.61 ± 0.45	2.80 ± 0.31	2.89 ± 0.38	3.11 ± 0.65	< 0.001
FT4 (ng/dL)	1.11 ± 0.24	0.92 ± 0.11	0.81 ± 0.11	0.68 ± 0.16	< 0.001
TSH (μIU/mL)	1.62 ± 1.96	1.58 ± 1.20	1.86 ± 3.68	3.52 ± 9.66	0.001
FT3/FT4 ratio	2.37 ± 0.29	3.06 ± 0.16	3.59 ± 0.16	4.68 ± 1.00	< 0.001

*SPPB* short physical performance battery. Values are presented as mean ± standard deviation for continuous variables, and *n* (%) for categorical variables

Among diseases only diabetes showed higher prevalence in the FT3/FT4 ratio lowest quartile of distribution (*p* = 0.002), but no association could be found with other THs.

No differences in the frequency of corticosteroid, anti-hypertensive, inotropic drugs, and methimazole use were found according to THs quartile of distribution (data not shown). Levothyroxine was prescribed, more often to cases in the highest quartile of TSH, compared to all others (10.6% vs 2.3%; *p* = 0.003, respectively). No association with other THs was found for any drug considered.

In the multivariate analysis, where SPPB score, strength, and muscle mass/height squared ratio were analyzed in different models, according to dummy variables derived from FT3, FT4, TSH, and FT3/FT4-ratios for quartile of distribution, and adjusted for age and sex, no changes in the significant differences were found (Table 5).

**Table 5:** Linear regression analysis, associations of physical performance (SPPB score), strength (handgrip), and muscle mass/height squared ratio with quartiles of distribution of thyroid hormones

	SPPB		Handgrip		Muscle/H^2^	
	*β* ± SE	*p* value	*β* ± SE	*p* value	*β* ± SE	*p* value
A						
Intercept	9.14 ± 1.48	< 0.001	61.09 ± 9.15	< 0.001	32.78 ± 8.56	< 0.001
TSH < 0.79	− 0.01 ± 0.14	0.95	0.33 ± 0.86	0.70	− 1.79 ± 0.78	0.02
TSH 0.80–1.25	0.05 ± 0.14	0.74	0.32 ± 0.86	0.71	− 1.64 ± 0.82	0.04
TSH 1.26–2.11	0.01 ± 0.14	0.99	1.01 ± 0.86	0.24	− 1.01 ± 0.78	0.20
TSH > 2.11	Reference					
Age (yy)	− 0.08 ± 0.02	< 0.001	− 0.45 ± 0.09	< 0.001	− 0.11 ± 0.09	0.25
Sex (female)	− 0.33 ± 0.11	0.004	− 8.33 ± 0.67	< 0.001	− 4.16 ± 0.62	< 0.001
B						
Intercept	8.96 ± 1.46	< 0.001	62.23 ± 9.11	< 0.001	31.17 ± 8.50	< 0.001
FT3 < 2.56	− 0.44 ± 0.14	0.002	− 0.38 ± 0.86	0.66	− 1.46 ± 0.80	0.07
FT3 2.57–2.78	− 0.29 ± 0.14	0.04	− 0.44 ± 0.88	0.62	− 0.67 ± 0.83	0.42
FT32.79–3.05	− 0.03 ± 0.14	0.84	− 0.68 ± 0.82	0.40	− 0.53 ± 0.78	0.50
FT3 > 3.05	Reference					
Age (yy)	− 0.08 ± 0.02	< 0.001	− 0.46 ± 0.10	< 0.001	− 0.09 ± 0.09	0.30
Sex (female)	− 0.33 ± 0.11	0.004	− 8.33 ± 0.67	< 0.001	− 4.01 ± 0.62	< 0.001
C						
Intercept	8.30 ± 1.48	< 0.001	58.21 ± 9.09	< 0.001	30.35 ± 8.61	< 0.001
FT4 < 0.73	0.44 ± 0.14	0.002	2.57 ± 0.85	0.003	0.78 ± 0.78	0.32
FT4 0.74–0.86	0.32 ± 0.14	0.02	1.62 ± 0.84	0.05	0.55 ± 0.79	0.49
FT4 0.75–1.00	0.09 ± 0.14	0.51	0.61 ± 0.86	0.48	− 0.42 ± 0.79	0.59
FT4 > 1.01	Reference					
Age (yy)	− 0.08 ± 0.02	< 0.001	− 0.43 ± 0.10	< 0.001	− 0.09 ± 0.09	0.30
Sex (female)	− 0.36 ± 0.11	0.002	− 8.45 ± 0.66	< 0.001	− 4.10 ± 0.63	< 0.001
D						
Intercept	7.98 ± 1.47	< 0.001	57.54 ± 9.20	< 0.001	19.48 ± 5.55	< 0.001
FT3/FT4 < 2.75	− 0.67 ± 0.14	< 0.001	− 1.95 ± 0.86	0.03	− 1.14 ± 0.51	0.02
FT3/FT4 2.76–3.32	− 0.31 ± 0.14	0.02	− 0.77 ± 0.84	0.35	− 1.07 ± 0.51	0.03
FT3/FT4 3.33–3.84	− 0.19 ± 0.14	0.17	0.34 ± 0.83	0.35	− 1.11 ± 0.51	0.03
FT3/FT4 > 3.84	Reference					
Age (yy)	− 0.07 ± 0.02	< 0.001	− 0.41 ± 0.10	< 0.001	− 0.06 ± 0.06	0.33
Sex (female)	− 0.35 ± 0.11	0.002	− 8.39 ± 0.66	< 0.001	− 1.71 ± 0.39	< 0.001

The lowest and the intermediate-lowest quartiles of TSH showed a reduction in muscle mass/height squared ratio, compared to the highest quartile (*β* ± SE − 1.79 ± 0.78; *p* = 0.02; *β* ± SE − 1.64 ± 0.82, respectively). No differences were found for physical performance and strength.

Similarly, lowest and intermediate-lowest FT3 quartiles showed a reduction in the SPPB score, compared to the upper quartile, independently from age and sex (*β* ± SE − 0.44 ± 0.14; *p* = 0.002; − 0.29 ± 0.14; *p* = 0.04, respectively). Physical performance (SPPB score) and strength (handgrip test) were associated with FT4 lower and intermediate-lower quartile, independent of age and sex. No differences among the four quartiles were detectable for muscle mass/height squared ratio.

The analysis for the FT3/FT4 ratio confirmed that muscle mass/height squared ratio was lower in low, intermediate-low, and intermediate-high compared to the highest quartile. For physical performance, differences were observed among low and intermediate-low compared to the highest quartile. Finally, in order to evaluate the independent but concurrent role of different markers of thyroid signaling hormones on physical performance, strength, and muscle mass, three separate linear regression models were analyzed (Table 6 parts 1, 2, and 3).

**Table 6:** Backward multiple linear regression analysis assessing the association between physical performance, muscle strength, and muscle mass/height squared ratio and thyroid hormone signaling

	Model A		Model B	
	*β* ± SE	*p* value	*β* ± SE	*p* value
1. SPPB score				
Intercept	7.73 ± 1.49	< 0.001	7.70 ± 1.49	< 0.001
TSH < 0.79 (lowest quartile)	0.01 ± 0.11	0.97		
TSH > 0.79	Reference			
FT3 < 2.56 (lowest quartile)	− 0.25 ± 0.12	0.03		
FT3 > 2.56	Reference			
FT4 < 0.73 (lowest quartile)	0.21 ± 0.12	0.09		
FT4 > 0.73	Reference			
FT3/FT4 < 2.75 (lowest quartile)	− 0.35 ± 0.13	0.001	− 0.49 ± 0.12	< 0.001
FT3/FT4 > 2.75	Reference			
*R*^2^	0.14		0.13	
AIC	− 0.900		− 0.544	
RSSE	395.5		398.7	
2. Handgrip test				
Intercept	57.94 ± 9.31	< 0.001	57.71 ± 9.13	< 0.001
TSH < 0.79 (lowest quartile)	0.07 ± 0.71	0.92		
TSH > 0.79	Reference			
FT3 < 2.56 (lowest quartile)	0.38 ± 0.76	0.62		
FT3 > 2.56	Reference			
FT4 < 0.73 (lowest quartile)	1.42 ± 0.73	0.05	1.78 ± 0.68	0.009
FT4 > 0.73	Reference			
FT3/FT4 < 2.75 (lowest quartile)	− 1.13 ± 0.84	0.18		
FT3/FT4 > 2.75	Reference			
*R*^2^	0.38		0.38	
AIC	1248.4		1244.3	
RSSE	11,131		11,189	
3. Muscle mass/height squared ratio				
Intercept	19.01 ± 5.63	0.001	19.04 ± 5.40	< 0.001
TSH < 0.79 (lowest quartile)	− 0.67 ± 0.41	0.09		
TSH > 0.79	Reference			
FT3 < 2.56 (lowest quartile)	− 0.48 ± 0.43	0.26		
FT3> 2.56	Reference			
FT4 > 1.01 (lowest quartile)	− 0.32 ± 0.52	0.54		
FT4 < 1.01	Reference			
FT3/FT4 > 3.84 (highest quartile)	1.20 ± 0.55	0.03	1.11 ± 0.42	0.009
FT3/FT4 < 3.84	Reference			
*R*^2^	0.11		0.10	
AIC	621.3		619.2	
RSSE	2380.5		2419.5	

In all the models, thyroid hormones were dummy categorized and were adjusted for age, sex, diabetes, and levothyroxine therapy and were not excluded from the analysis even if they did not reach a statically significant level. Model A saturated model; Model B parsimonious model

The lowest quartile of FT3/FT4 ratio, independent of age and sex, was the only marker associated with SPPB, accounting for 8% of the 14% of the total variance explained by the model. Moreover, subjects in this lowest quartile (FT3/FT4 ratio < 2.75) showed a mean reduction of physical performance of 0.50 points, compared to all other groups (Table 6 part 1). In the model that analyzes handgrip, lowest quartile of FT4 (values < 0.73 mg/mL) was associated with a significant increase in strength (Table 6 part 2). Muscle mass/height squared ratio was higher, in the highest quartile (values > 3.84) of the FT3/FT4 ratio (*β* ± SE = 1.11 ± 0.42; *p* = 0.009), compared to all other groups (Table 6 part 3). All the models were adjusted for age, sex, diabetes, levothyroxine prescription, and the other THs.

## Discussion

This study demonstrates that in a cohort of free-living nonagenarian subjects, several measures of physical performance and muscle fitness are related to thyroid hormone levels. Lower free T3/free T4 ratio is associated with lower SPPB score, and the highest quartile of FT4 with a higher handgrip test result, whereas the lowest FT3/FT4 quartile is correlated with higher muscle mass. All these associations were assessed in models demonstrating their independence from potential confounding factors.

The major finding of this study is the association of low FT3/FT4 ratio and low physical performance, independent from the concurrent effect of the other THs, in an oldest-old population, bridging the gap of available data.

In the InCHIANTI study, after adjusting for multiple confounders, an association between physical performance and FT3, but not FT4 or TSH, was found [24]. The populations of the InCHIANTI and the Mugello studies were selected in the same region (Tuscany), but in the latter, it is represented only by nonagenarians; the two studies differed also in design, i.e., a cross-sectional design for the Mugello study and a longitudinal one for the InCHIANTI. Moreover, the FT3/FT4 ratio was not analyzed in the InCHIANTI results. Ceresini et al. found a threefold increase in the risk of impaired mobility (defined by SPPB < 9) in subjects of the InCHIANTI study with subclinical hyperthyroidism [25]. In a smaller study of fifty-one elderly fit subjects, levels of thyroid hormones (FT3/FT4 ratio) were correlated with aerobic endurance capacity and strength [26]. Recently, Kong et al., in a Korean population aged 40–69 years, demonstrated that a low FT3/FT4 ratio, but not serum FT3 or FT4 alone, was associated with low muscle mass and impaired physical performance [15]. Serum TSH level was shown to be inversely correlated with handgrip strength in a large cross-sectional study in Germany [27]. Other studies failed to detect a relationship between thyrotropin and performance in elderly subjects [28]. Recently, two cross-sectional studies of the same cohort of centenarians demonstrated that THs were associated with frailty, disability, and cognitive status [13, 16]. Moreover, both the increase of FT4 and the decrease of the FT3/FT4 ratio were associated with lower survival [13]. These data are consistent with the results of our study, since lower level of physical performance, muscle mass reduction, and lower muscle strength are components of the frailty phenotype [29]. In addition, a decrease of the SPPB score is a strong predictor of subsequent disability [2] and also of catastrophic events such as institutionalization or death [22].

Skeletal muscle is a target of thyroid hormone signaling, acting on muscle contractility and metabolism, by regulating gene expression in an age-dependent manner [30]. Thyroid hormone–converting enzymes (DIO2 and DIO3) locally control the uptake and the activation or inactivation of TH within the skeletal muscle tissue. Muscle gene expression, phenotype, plasticity, energy turnover, and glucose metabolism are controlled and regulated in a T3-dependent fashion [14]. During aging, changes occurring in the thyroid structure and function affect TH production, metabolism, transport, and action; tissue-specific regulation of deiodinase activities occurs with aging, but TH signaling in the muscle, heart, and brain appears to be unaltered [31].

Recently Franceschi et al., reviewing the complex interrelationship among aging, longevity, and thyroid aging, highlighted how complex and heterogeneous are those traits interactions, particularly in the oldest-old [32]. Moreover, they suggested that similar to inflammaging or to osteoporosis, also those thyroid age–related changes could be part of the “systemic adaptive remodeling” that had the function to prevent or mitigate processes of tissue disruption and degenerative changes [33].

Therefore, our results need to be evaluated keeping in mind those two postulates. First, the cross-sectional character of the study does not consider the interaction of lifelong internal and external factors leading to individual heterogeneity. Second, changes occurring during thyroid aging might be considered adaptive, and not only detrimental.

Collectively this data indicate the FT3/FT4 ratio as a possible marker of muscle aging, even if the action of THs on several target organs and their functions may play a role in modulating physical performance and strength.

To the best of our knowledge, this is the first study assessing the concurrent role of the different THs in a large population of nonagenarians, and showing the relationship of the FT3/FT4 ratio with aging muscle function.

Among the limitations due to the cross-sectional design, this study could only detect associations between TH and markers of muscle aging, and not a causal relationship. Recall bias, frequent in elderly subjects in the Mugello study were overcome with the required presence of the caregiver during the clinical session. Moreover, as suggested by Maggio et al., individual hormones do not operate independent of each other; rather, one hormonal problem may trigger the onset of another [34, 35], such that associations between individual hormones and health status may result from a wider hormonal dysfunction or from concurrent effects on other physiological systems.

## Conclusion

In a population of (free-living) nonagenarians, levels of thyroid hormones have been shown to play a central role in the variation of physical performance and muscle strength, which are key markers of the aging process. We could not establish whether the FT3/FT4 ratio may be a marker of successful aging or just an epiphenomenon of the aging muscle. Longitudinal evaluations are required to verify these findings and elucidate the pathway(s) linking thyroid and muscle function in aging.

## Data Availability

The Mugello study dataset is not stored in a data repository, but data is available on reasonable request.
